# Alleviative Effect of Alpha-Lipoic Acid on Cognitive Impairment in High-Fat Diet and Streptozotocin-Induced Type 2 Diabetic Rats

**DOI:** 10.3389/fnagi.2021.774477

**Published:** 2021-11-12

**Authors:** Chih-Yuan Ko, Jian-Hua Xu, Yangming Martin Lo, Rong-Syuan Tu, James Swi-Bea Wu, Wen-Chung Huang, Szu-Chuan Shen

**Affiliations:** ^1^Department of Clinical Nutrition, The Second Affiliated Hospital of Fujian Medical University, Quanzhou, China; ^2^Department of Respiratory and Critical Care Medicine, The Second Affiliated Hospital of Fujian Medical University, Quanzhou, China; ^3^School of Public Health, Fujian Medical University, Fuzhou, China; ^4^Respiratory Medicine Center of Fujian Province, Quanzhou, China; ^5^Department of Tumor Surgery, The Second Affiliated Hospital of Fujian Medical University, Quanzhou, China; ^6^Institute for Advanced Study, Shenzhen University, Shenzhen, China; ^7^Graduate Program of Nutrition Science, National Taiwan Normal University, Taipei City, Taiwan; ^8^Graduate Institute of Food Science and Technology, National Taiwan University, Taipei City, Taiwan; ^9^Graduate Institute of Health Industry Technology, Chang Gung University of Science and Technology, Taoyuan City, Taiwan

**Keywords:** α-lipoic acid, Alzheimer’s disease, cerebral insulin signaling, long-term potentiation, type 2 diabetes

## Abstract

**Background:** The intricate relationship between type 2 diabetes mellitus (T2DM) and Alzheimer’s disease (AD) suggests that insulin is involved in modulating AD-related proteins. Alpha-lipoic acid (ALA) can improve insulin resistance (IR) in diabetic rats. However, the role of ALA in alleviating the cognitive decline of T2DM is not yet clear. This study examined the ameliorative effect of ALA on cognitive impairment, cerebral IR, and synaptic plasticity abnormalities in high-fat diet (HFD) plus streptozotocin (STZ) induced diabetic rats.

**Methods:** The HFD/STZ-induced T2DM male Wistar rats were orally administered with ALA (50, 100, or 200 mg/kg BW) once a day for 13 weeks. Abilities of cognition were measured with a passive avoidance test and Morris water maze. Specimens of blood and brain were collected for biochemical analysis after the rats were sacrificed. Western blotting was used to determine protein expressions in the hippocampus and cortex in the insulin signaling pathways, long-term potentiation (LTP), and synaptic plasticity-related protein expressions.

**Results:** Alpha-lipoic acid improved hyperinsulinemia and the higher levels of free fatty acids of the T2DM rats. Behavioral experiments showed that the administration of ALA improved cognitive impairment in HFD/STZ-induced T2DM rats. ALA ameliorated insulin-related pathway proteins [phosphoinositide 3-kinase (PI3K), phospho-protein kinase B (pAkt)/Akt, and insulin-degrading enzyme (IDE)] and the LTP pathway, as well as synaptic plasticity proteins (calmodulin-dependent protein kinase II, cyclic AMP response element-binding protein, and postsynaptic density protein-95) of the cerebral cortex or hippocampus in HFD/STZ-induced T2DM rats.

**Conclusion:** Our findings suggested that ALA may ameliorate cognition impairment *via* alleviating cerebral IR improvement and cerebral synaptic plasticity in diabetic rats.

## Introduction

Obesity poses a risk factor for type 2 diabetes mellitus (T2DM). Not only does obesity induce metabolic disorders and insulin resistance (IR), but it also promotes β-cell dysfunction (Eckel et al., 2011). IR is the most prominent symptom of T2DM. When cellular IR occurs, glucose in the blood cannot be effectively used, and the body is in a state of high blood sugar and high insulin for a long time, which leads to an increase in oxidative stress in the body and subsequently causes the body to produce a chronic inflammatory response. Additionally, IR in peripheral tissues can also cause IR in the brain. The role of insulin in the brain can affect neuron development, synapse formation, learning, memory, and glucose regulation (Derakhshan and Toth, 2013). Evidence has demonstrated that T2DM is a risk factor for the development of Alzheimer’s disease (AD) or dementia (Chatterjee and Mudher, 2018).

The brain is an organ with high sensitivity to insulin. The functions of brain can be regulated by insulin including neuron development, synapse formation, learning and memory, glucose regulation, and feeding behavior (Derakhshan and Toth, 2013). It has been verified that insulin can penetrate the blood-brain barrier (BBB) through a transporter, consequently regulating the relevant information transmission pathways in the brain (Derakhshan and Toth, 2013). Insulin receptors are transmembrane receptors that are present in the cerebral hippocampus and cortex that play a critical role in the formation of learning memory (Cole et al., 2007). Decreased insulin and related receptor activities were demonstrated with brain IR in postmortem AD brains (Talbot et al., 2012).

Alzheimer’s disease causes nerve cell death and brain tissue atrophy; however, the cause of the disease is still not clarified. Plaque deposits formed by β-amyloid (Aβ) and neurofibrillary tangles (NFTs) due to the hyperphosphorylation of Tau proteins are well-known hypothesized causes, since they are considered the main pathological feature of AD (Bloom, 2014). Additionally, hypotheses regarding IR, inflammation, oxidative imbalance, and gene mutations have been proposed (Gu et al., 2016). When generating IR, the main PI3K/protein kinase B (Akt/PKB) of the insulin signal pathway loses activity so that blood sugar cannot be effectively regulated. Meanwhile, glycogen synthase kinase 3β (GSK-3β) is activated. GSK-3β is a key step to increasing phosphorylation of Tau proteins. When Tau proteins are over phosphorylated, they are separated from microtubules and can eventually be aggregated to form NFTs (Špolcová et al., 2014). Moreover, protein glycation is the course and complication of diabetes. Depending on the pathology, Tau protein glycosylation induces the formation of NFTs in the development of AD (Chatterjee and Mudher, 2018).

The brain can scavenge Aβ by various mechanisms, such as protein degradation, glial cell phagocytosis, and transport out of brain nerve cells through the BBB (Ries and Sastre, 2016). However, when IR or hyperinsulinemia occurs, IDEs can degrade and clear Aβ in brain. Insulin and Aβ compete with each other for the IDEs of the brain. Due to the high affinity of insulin and IDEs, Aβ cannot be removed and thus accumulates in the brain prompting further AD pathogenesis (Son et al., 2016).

Moreover, decline of cognitive functions and gradual loss of memory are AD features. The hippocampus serves an important function in learning and memory. Presynaptic neurons mainly use glutamate as a neurotransmitter to connect *N*-methyl-D-aspartate receptors (NMDARs) and non-NMDARs (also known as AMPARs) on postsynaptic neurons. When short-term high-frequency stimulation is given, the postsynaptic potentials increase, and Mg^2+^ on the NMDAR can be repelled to open the channel at this time. When Ca^2+^ enters the synapse and activates the cascade reaction of calmodulin and Ca^2+^/calmodulin-dependent protein kinase II (CaMKII), long-term potentiation (LTP) is formed (Nicoll, 2017). Aβ and its active fragments can inhibit LTP induced by high-frequency stimulation in the hippocampus of rats. However, the complex mechanisms of hippocampal LTP are not yet entirely understood.

Alpha-lipoic acid (ALA), a natural antioxidant, is widely observed in various plants and animals. Studies have found that it has an anti-inflammatory effect, prevents vascular diseases, reduces cognitive impairment, and alleviates diabetes and related complications (Shay et al., 2009; Fava et al., 2013; Liu et al., 2017; Zhang et al., 2018; Ko et al., 2021). The present study aimed to explore the effects of ALA on improving cognitive impairment, brain IR, and abnormal synaptic plasticity using a high-fat diet (HFD) plus streptozotocin (STZ)-induced T2DM model rats.

## Materials and Methods

### Animal Experimental Procedures

Animal experimental procedures used in this study were adapted from our previous study (Ko et al., 2021). Eight-week-old male Wistar rats were reared in a temperature- (22 ± 1°C) and humidity-controlled (50 ± 20%) room, under a 12-h light/dark cycle (lights on from 08:00 to 20:00), with free access to food and water in the animal room of Taiwan Normal University. The study was approved by the Institutional Animal Care and Use Committee of National Taiwan Normal University with the certificate number 106042.

After adapting to the environment for 1 week, rats were fed a HFD (60% calories from fat) or a normal diet for 4 weeks. HFD-fed rats were then intraperitoneally injected with STZ (Sigma, St. Louis, MO, United States) [30 mg/kg body weight (BW)] to induce diabetes, and then were fed with a HFD for nine more weeks. Thirty-six rats were randomly divided into six groups with six rats in each. The normal group contained rats fed with a normal diet; the DM group contained diabetic rats fed with a HFD alone as the negative control; the DM + PIO group contained diabetic rats fed with a HFD and orally administered pioglitazone (PIO, 30 mg/kg BW) daily for 13 weeks as the positive control; and the DM + ALA50, DM + ALA100, and DM + ALA200 groups, which contained diabetic rats daily fed with a HFD and orally administered 50, 100, or 200 mg/kg BW ALA (Sigma, St. Louis, MO, United States), respectively, for 13 weeks. The dosages used were the same as in our previous study (Ko et al., 2021). All rats were sacrificed at the end of the experiment.

### Passive Avoidance Test

The passive avoidance test was slightly modified from a previous study (Elrod and Buccafusco, 1988). Basically, all test subjects were divided into separate rooms with a gate in the middle of the two rooms and an electric shock net at the bottom of the two rooms, but only the electric shock net in the dark room was energized. Before the experiment, the rats were acclimatized in the dark room for 15 min and then the experiment was started.

The first day was the training period. The rat entered from the bright room. When it entered the darkroom completely through the gate, the gate was shut and the rat was given an electric shock for 2 s. After the electric shock was over, the rat stayed in the dark room for f 5 s, and then was put back into the breeding cage before taking records. Rats completed the learning and training within the latency time of the bright room. If the rat had not entered the darkroom within 300 s, it was forced to enter the dark room and an electric shock was given to complete the learning and training process. After completing the learning and training, the next continuous test began and lasted for 3 days, with the exception that at this time, the test subject did not receive any electric shocks. The time the rats stayed in the bright room was recorded, since this test enabled observation of the rat’s ability to continue learning and memory.

### Morris Water Maze Test

The MWM protocol was adopted from previous studies (Morris, 1984; Vorhees and Williams, 2006). For spatial acquisition, rats performed spatial learning in four separate quadrants every day, and the experiment was repeated for a total of five consecutive days. The rat was placed facing the wall of the pool at the starting position of each quadrant. When the rat was positioned in the water, timing began immediately, and when the rat climbed (touched) the platform, the time was stopped and recorded. The duration of one test was approximately 2 min. If the rat did not reach the underwater platform within 2 min, it was led to face the east and placed on the platform for 15 s. Once the test was completed, another animal was employed for the test in the same quadrant. After all animals had completed the test in the first quadrant, the tests were repeated in the second quadrant until all four quadrants were covered.

To probe the trial measurement, after 24 h of the positioning navigation test, the target platform in the water was removed, and the rat was placed facing the wall of the pool in the relative position of the original target platform before timing started. The time was recorded when passing the original target platform position for the first time. If the time exceeded 30 s, the rat was removed from the water maze.

For a physical function test, the platform was placed in the new quadrant and exposed 2 cm in height above the water surface to make it a visible platform. This test was used to confirm that the rat’s visual movement function was normal, and the time was recorded when the rat found the platform.

### Tissue Protein Preparation and Measurement

To prepare brain protein samples, 100 mg of the cerebral cortex or hippocampus were mixed with 0.5 mL lysis buffer and ground three times (10 s each time). After 1-h incubation in an ice bath, the homogenized tissue solutions were centrifuged at 20,000 × *g* under 4°C for 1 h to obtain tissue protein supernatants. The protein concentration was measured at 595 nm using the Bradford method with a Bio-Rad protein assay kit (Hercules, CA, United States).

### Western Blot

Aliquots of brain tissue protein samples, each containing 40 μg of protein, were evaluated for the expression of insulin receptor substrate 1 (IRS-1), PI3K, Akt/PKB, pAkt, GSK-3β, pGSK-3β, IDE, pTau (Thr181), Tau, *N*-methyl-D-aspartate receptor (NMDAR)1, NMDAR2β, calmodulin, CaMK II, cyclic AMP response element (CRE)-binding protein (CREB), postsynaptic density protein 95 (PSD-95), and brain derived neurotrophic factor (BDNF). The samples were subjected to 10% sodium dodecyl sulfate-polyacrylamide gel electrophoresis. The separated proteins were electrotransferred to a polyvinylidene difluoride membrane. The membrane was incubated with blocking buffer [phosphate-buffered saline (PBS) containing 0.05% Tween-20 (PBST) and 5% (wt/vol) non-fat dry milk] for 1 h, incubated overnight at 4°C with PBST, and probed with anti-IRS-1 (1906-7), anti-PI3K (60225-1-lg), anti-AKT (9272), anti-pAKT (9271), anti-GSK3β (9315), anti-pGSK3β (9336), anti-Tau (4019), anti-CaMKII (44436), anti-CREB (9197), and anti-IDE (67106-1-lg) antibodies (1:1000; Signalway Antibody, MD, United States), anti-PSD95 (ab18258) and anti-BDNF (ab108383) antibodies (1:1000; Abcam, MA, United States) as well as anti-pTau (GTX50171), anti-NMDAR1 (GTX133097), anti-NMDAR 2β (GTX133099), and anti-calmodulin (GTX52363) antibodies (1:1000; GeneTex, Hsinchu, Taiwan). A 1:5000 dilution of mouse monoclonal α-tubulin (3873) or GAPDH (5174) antibodies (Signalway Antibody, MD, United States) were used to ensure that a constant amount of protein was loaded into each lane of the gel. The membrane was washed three times (5 min each) in PBST, shaken in a solution of horseradish peroxidase-conjugated anti-mouse IgG or anti-rabbit IgG (Genetex, Irvine, CA, United States) secondary, washed three times (5 min each) in PBST, and incubated in enhanced chemiluminescence reagent (Millipore, Darmstadt, Germany). Autoradiography was performed and analyzed using a UVP Biospectrum image system (Level, Cambridge, United Kingdom). Finally, all relevant protein expressions were normalized with α-tubulin or GAPDH.

### Blood Samples Collection

Rats were fasted overnight (8-h) and blood was sampled from the tail vessels before they were sacrificed. Blood was collected and centrifuged at 12,000 × *g* for 8 min to obtain the serum samples. The samples were stored at −80°C before use.

### Measurement of Serum Insulin and Plasma Free Fatty Acid

Levels of insulin and FFA were measured by the commercial enzyme-linked immunosorbent assay (ELISA) kits (Mercodia AB and Uppsala, Sweden; Crumlin, Co., Antrim, United Kingdom). Biochemical analyses were conducted according to the supplier’s protocols.

### Statistical Analyses

Values are presented as the mean ± standard error of the mean using SPSS version 22.0 (SPSS Inc., Chicago, IL, United States). A one-way analysis of variance (ANOVA) and Duncan’s multiple range tests were performed. *P* < 0.05 was accepted as statistically significant.

## Results

### Effects of Alpha-Lipoic Acid on High-Fat Diet/Streptozotocin-Induced Brain Tissue Weight and Serum Insulin in Type 2 Diabetes Mellitus Rats

Serum insulin of the DM group was significantly higher than that of the DM + PIO and ALA groups (Table 1). The plasma free fatty acid of the DM group was significantly higher than that of the normal, DM + PIO, and ALA200 groups (Table 1). The brain tissue weight of the DM group was significantly lower than that of the normal and ALA200 groups (Table 1).

**TABLE 1:** Profiles of fasting insulin, free fatty acid, and brain weight in high-fat diet and streptozotocin-induced type 2 diabetic rats treated with alpha-lipoic acid for 13 weeks.

	Normal	DM + PIO	DM	DM + ALA50	DM + ALA100	DM + ALA200
Fasting serum insulin (μg/L)	1.020.15^ab^	0.660.06^b^	1.240.23^a^	0.720.14^b^	0.690.03^b^	0.720.07^b^
Fasting plasma free fatty acid (mmol/L)	0.650.05^b^	0.770.06^b^	1.170.07^a^	0.890.03^*ab*^	0.860.04^ab^	0.570.02^b^
Brain weight (g)	1.640.02^a^	1.590.02^ab^	1.490.05^b^	1.560.03^ab^	1.610.03^ab^	1.630.02^a^

*Normal, Normal diet; DM, high-fat diet (HFD; 60% fat) with STZ (30 mg/kg body weight, i.p.) induced diabetic rats; DM + Pio, DM rats gavaged with pioglitazone (30 mg/kg body weight) for 13 weeks; DM + ALA50, DM rats gavaged with ALA (50 mg/kg body weight) for 13 weeks; DM + ALA100, DM rats gavaged with ALA (100 mg/kg body weight) for 13 weeks; DM + ALA200, DM rats gavaged with ALA (200 mg/kg body weight) for 13 weeks.*

*Values were calculated as the mean ± SEM, *n* = 6 for each group.^*a,b*^letter is significantly different among all samples tested (*p* < 0.05).*

### Passive Avoidance Test

**FIGURE 1:**
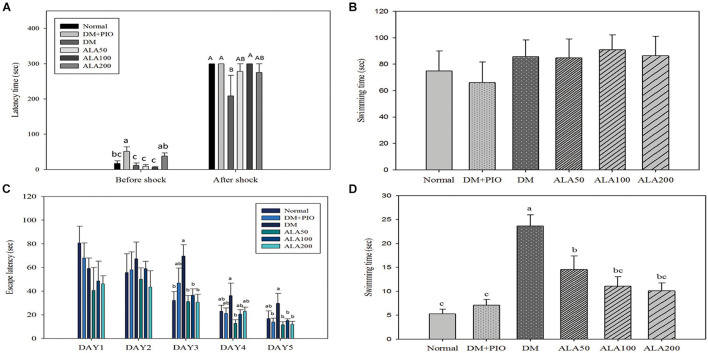
Passive avoidance test **(A)**, physical function **(B)**, spatial acquisition **(C)**, and probe trial **(D)** of Morris Water Maze of high-fat diet and streptozotocin-induced type 2 diabetic rats treated with alpha-lipoic acid for 13 weeks. Normal, Normal diet; DM, high-fat diet (HFD; 60% fat) with STZ (30 mg/kg body weight, i.p.) induced diabetic rats; DM + Pio, DM rats gavaged with pioglitazone (30 mg/kg body weight) for 13 weeks; DM + ALA50, DM rats gavaged with ALA (50 mg/kg body weight) for 13 weeks; DM + ALA100, DM rats gavaged with ALA (100 mg/kg body weight) for 13 weeks; DM + ALA200, DM rats gavaged with ALA (200 mg/kg body weight) for 13 weeks. Values were calculated as the mean ± SEM, *n* = 6 for each group. a–c letter is significantly different among all samples tested before the shock of the passive avoidance test or Morris Water Maze test (*p* < 0.05). A, B letter is significantly different among all samples tested in after shock of passive avoidance test (*p* < 0.05).

The passive avoidance test is an experimental method that utilizes a darkening environment for the rats and was utilized to observe rats’ abilities in learning memory. The results from the before-shock experiments showed that when the experimental animals in each experimental group entered the darkroom, the DM + PIO group remained significantly longer than the normal, DM, ALA50, and ALA100 groups (Figure 1A). Contrarily, the after-shock results showed that the normal, DM + PIO, and ALA100 groups stayed in the bright room for a significantly longer time than the DM group (Figure 1A).

### Morris Water Maze

The MWM test is the most commonly used method to assess the spatial learning and memory abilities of rodents (Morris, 1984). For the physical function of MWM, there was no difference in the basic physiological functions (visual and motor abilities) of all rats (Figure 1B).

There was no statistical difference in the results between each group on the first and second days of the spatial acquisition test (Figure 1C). On the third day of the test, the DM group took significantly more time looking for a platform in the water than the normal, ALA50, ALA100, and ALA200 groups (Figure 1C). On the fourth day of the test, only the ALA50 and DM groups had a significant difference. Compared with other groups, the DM group had an increase in time, but there was no statistically significant difference (Figure 1C). The results on the last day of the trial showed that the DM group (29.70 ± 8.52 s) exhibited a significantly longer time than the DM + PIO, ALA50, ALA100, and ALA200 groups (Figure 1C). The probe trial of MWM was to evaluate the reference memory after the learning experience (Vorhees and Williams, 2006). For the probe trial of MWM, the DM group took a significantly longer time than the other groups (Figure 1D).

### The Effect of Alpha-Lipoic Acid on the Insulin Signal Pathway in the Cerebral Hippocampus and Cortex in Type 2 Diabetes Mellitus Rats and the Expression of Proteins Related to the Phosphorylation Pathway of Tau Protein

**FIGURE 2:**
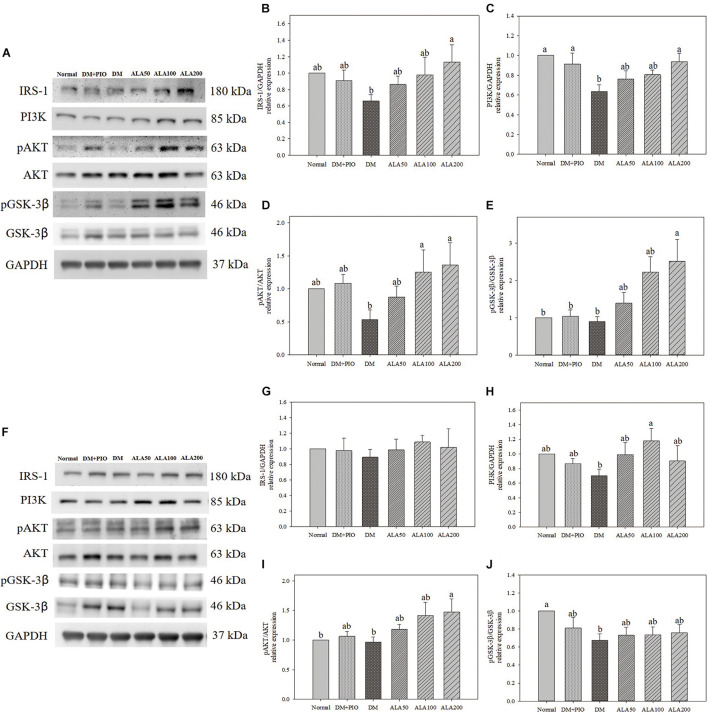
Protein expression levels of the hippocampus **(A)** and cortex **(F)** in quantifying relative IRS-1 **(B,G)**, PI3K **(C,H)**, pAKT/AKT **(D,I)**, and pGSK-3β/GSK-3β **(E,J)** in high-fat diet and streptozotocin-induced type 2 diabetic rats treated with alpha-lipoic acid for 13 weeks. Normal, Normal diet; DM, high-fat diet (HFD; 60% fat) with STZ (30 mg/kg body weight, i.p.) induced diabetic rats; DM + Pio, DM rats gavaged with pioglitazone (30 mg/kg body weight) for 13 weeks; DM + ALA50, DM rats gavaged with ALA (50 mg/kg body weight) for 13 weeks; DM + ALA100, DM rats gavaged with ALA (100 mg/kg body weight) for 13 weeks; DM + ALA200, DM rats gavaged with ALA (200 mg/kg body weight) for 13 weeks. Values were calculated as the mean ± SEM, *n* = 6 for each group. a,b letters indicate significant differences among all samples tested (*p* < 0.05).

Effects of ALA on expression of proteins of the insulin signal pathway in the cerebral hippocampus (Figure 2A) and cortex (Figure 2F). The expression of IRS-1 protein in the hippocampus of the ALA200 group was significantly higher than that of the DM group, an increase of 71.5% (Figure 2B). There was no statistical difference in the expression level of cortical IRS-1 protein among the groups (Figure 2G).

The expression levels of PI3K in the hippocampus of normal, DM + PIO, and ALA200 groups was significantly higher than that of the DM group (Figure 2C). The expression level of PI3K in the cortical DM group was lower than that of others, but it was only significantly different from the ALA100 group, which was a significant increase of 68.6% (Figure 2H).

The expression levels of pAKT/AKT in the hippocampus of the ALA100 and ALA200 groups was significantly higher than that of the DM group (Figure 2D). The ALA200 group of the cortex was significantly higher than in the normal and DM groups (Figure 2I).

The expression levels of pGSK-3β/GSK-3β in the hippocampus of the ALA200 group was significantly higher than that of the normal, DM + PIO, and DM groups (Figure 2E). The normal group was significantly higher in the cortex than the DM group (Figure 2J).

**FIGURE 3:**
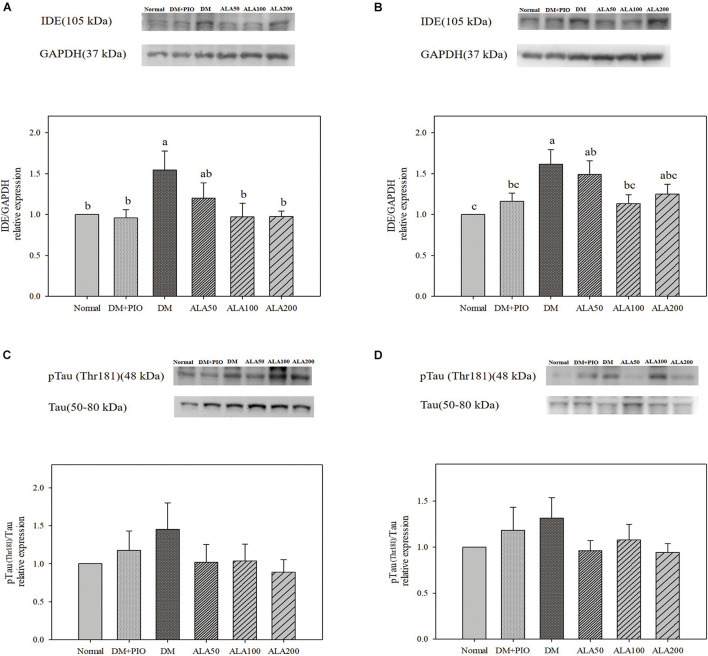
Protein levels of IDE in the hippocampus **(A)** and cortex **(B)**, and pTau (Thr181)/Tau levels in the hippocampus **(C)** and cortex **(D)** in high-fat diet and streptozotocin-induced type 2 diabetic rats treated with alpha-lipoic acid for 13 weeks. Normal, Normal diet; DM, high-fat diet (HFD; 60% fat) with STZ (30 mg/kg body weight, i.p.) induced diabetic rats; DM + Pio, DM rats gavaged with pioglitazone (30 mg/kg body weight) for 13 weeks; DM + ALA50, DM rats gavaged with ALA (50 mg/kg body weight) for 13 weeks; DM + ALA100, DM rats gavaged with ALA (100 mg/kg body weight) for 13 weeks; DM + ALA200, DM rats gavaged with ALA (200 mg/kg body weight) for 13 weeks. Values were calculated as the mean ± SEM, *n* = 6 for each group. a–c letter is significantly different among all samples tested (*p* < 0.05).

The expression levels of IDE in the hippocampus of the DM group was significantly higher than that of the normal, DM + PIO, ALA100, and ALA200 groups (Figure 3A). The DM group was significantly higher in the cortex than the normal, DM + PIO, and ALA100 groups (Figure 3B). However, the expression of pTau (Thr181)/Tau protein in the hippocampus (Figure 3C) and cortex (Figure 3D) of each group did not reach a statistically significant difference.

### The Effect of Alpha-Lipoic Acid on the Expression of Proteins Related to Long-Term Potentiation Related Pathways in the Cerebral Hippocampus and Cortex in Type 2 Diabetes Mellitus Rats

**FIGURE 4:**
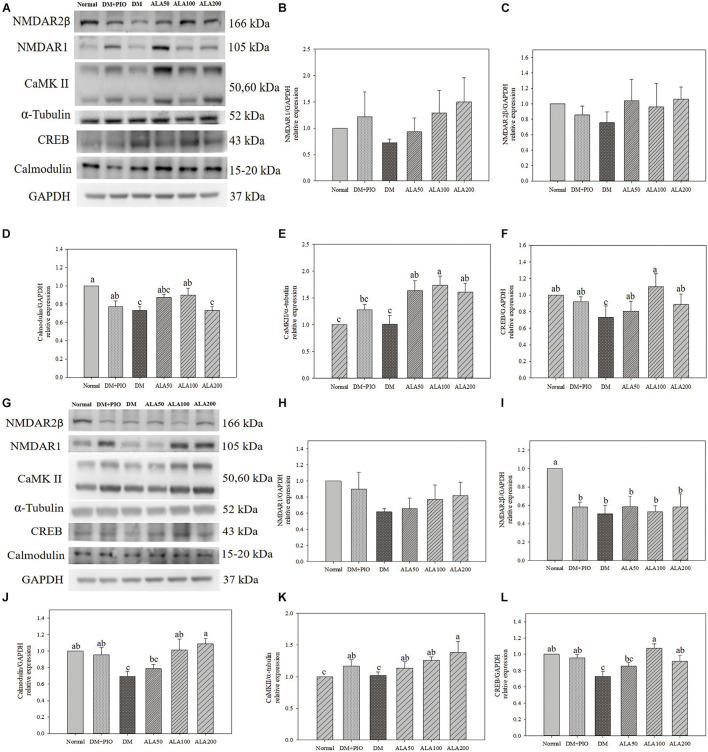
Protein expression levels of the hippocampus **(A)** and cortex **(G)** in quantifying relative NMDAR1 **(B,H)**, NMDAR2β **(C,I)**, calmodulin **(D,J)**, CaMK II **(E,K)**, and CREB **(F,L)** in high-fat diet and streptozotocin-induced type 2 diabetic rats treated with alpha-lipoic acid for 13 weeks. Normal, Normal diet; DM, high-fat diet (HFD; 60% fat) with STZ (30 mg/kg body weight, i.p.) induced diabetic rats; DM + Pio, DM rats gavaged with pioglitazone (30 mg/kg body weight) for 13 weeks; DM + ALA50, DM rats gavaged with ALA (50 mg/kg body weight) for 13 weeks; DM + ALA100, DM rats gavaged with ALA (100 mg/kg body weight) for 13 weeks; DM + ALA200, DM rats gavaged with ALA (200 mg/kg body weight) for 13 weeks. Values were calculated as the mean ± SEM, *n* = 6 for each group. a–c letter is significantly different among all samples tested (*p* < 0.05).

Effects of ALA on expression of proteins of LTP related pathways in the cerebral hippocampus (Figure 4A) and cortex (Figure 4G). The expression of NMDAR1 protein in the hippocampus (Figure 4B) and cortex (Figure 4H) of each group did not reach a statistically significant difference.

There was no statistical difference in the expression levels of hippocampus NMDAR2β protein among groups (Figure 4C). While the normal group was significantly higher in the cortex than the groups (Figure 4I).

The expression levels of calmodulin in the hippocampus of the normal group was significantly higher than that of the DM and ALA200 groups (Figure 4D). The DM group was significantly lower in the cortex than the normal, DM + PIO, ALA100, and ALA200 groups (Figure 4J).

There was no statistical difference in the expression levels of CaMK II in hippocampus between normal and DM groups, but the DM group was significantly lower than that of the ALA50, ALA100, and ALA200 groups (Figure 4E). The ALA200 group was significantly higher in the cortex than the normal and DM groups (Figure 4K).

The expression levels of CREB in the hippocampus of the DM group was significantly reduced by 33.6% compared with the ALA100 group (Figure 4F). The DM group was significantly lower in the cortex than the normal, DM + PIO, ALA100, and ALA200 groups (Figure 4L).

### The Effect of Alpha-Lipoic Acid on the Expression of Proteins Related to Synaptic Plasticity in the Cerebral Hippocampus and Cortex in Type 2 Diabetes Mellitus Rats

**FIGURE 5:**
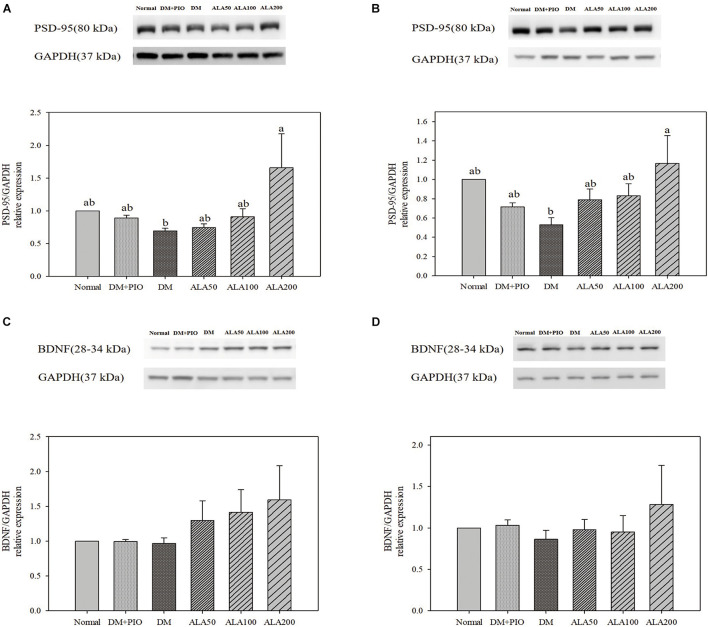
Protein levels of PSD-95 in the hippocampus **(A)** and cortex **(B)**, and BDNF levels in the hippocampus **(C)** and cortex **(D)** in high-fat diet and streptozotocin-induced type 2 diabetic rats treated with alpha-lipoic acid for 13 weeks. Normal, Normal diet; DM, high-fat diet (HFD; 60% fat) with STZ (30 mg/kg body weight, i.p.) induced diabetic rats; DM + Pio, DM rats gavaged with pioglitazone (30 mg/kg body weight) for 13 weeks; DM + ALA50, DM rats gavaged with ALA (50 mg/kg body weight) for 13 weeks; DM + ALA100, DM rats gavaged with ALA (100 mg/kg body weight) for 13 weeks; DM + ALA200, DM rats gavaged with ALA (200 mg/kg body weight) for 13 weeks. Values were calculated as the mean ± SEM, *n* = 6 for each group. a–c letter is significantly different among all samples tested (*p* < 0.05).

The expression levels of PSD-95 in the hippocampus (Figure 5A) and cortex (Figure 5B) of the ALA200 group was significantly higher than that of the DM group. However, the expression of BDNF protein in the hippocampus (Figure 5C) and cortex (Figure 5D) of each group did not reach a statistically significant difference.

## Discussion

**FIGURE 6:**
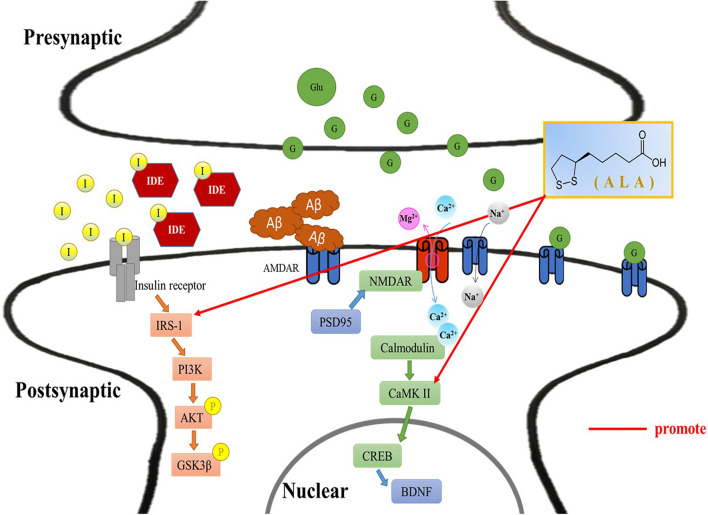
Alleviative mechanism of α-lipoic acid (ALA) in the hippocampus and cortex of T2DM rats induced by HFD/STZ. Postulated mechanism through which ALA alleviates insulin receptor substrate-1 and calmodulin-dependent protein kinase II in the hippocampus and cortex of high-fat diet and streptozotocin-induced type 2 diabetic rats. I, insulin; IDE, insulin-degrading enzyme; IRS-1, insulin receptor substrate 1; PI3K, phosphoinositide 3-kinase; Akt/PKB, protein kinase B; P, phosphorylation; GSK-3β, glycogen synthase kinase 3β; Aβ, β-amyloid; NMDAR, *N*-methyl-D-aspartate receptor; PSD-95, postsynaptic density protein 95; CaMKII, Ca^2+^/calmodulin-dependent protein kinase II; CREB, cyclic AMP response element-binding protein; BDNF, brain derived neurotrophic factor; Glu/G, glucose; ALA, α-lipoic acid.

In this study, ALA improves hyperinsulinemia and high levels of FFA, and slows brain atrophy caused by T2DM. Additionally, behavioral experiments demonstrated that ALA alleviates cognitive impairment in HFD/STZ-induced T2DM rats. This is the postulated mechanism through which ALA alleviates PI3K of the insulin signal pathway and regulates CaMKII of the LTP-related pathway in the hippocampus and cortex of HFD/STZ-induced T2DM rats (Figure 6).

Alpha-lipoic acid can act as a dietary supplement benefit for patients with DM (Shay et al., 2009). A clinical study revealed that ALA (600 mg/day) ameliorates cognitive decline in AD patients with DM (Fava et al., 2013). The underlying molecular mechanisms and therapeutic potential were not clear. IR is deemed to be a common risk factor for DM and AD (Ott et al., 1999). Chronic overnutrition and obesity are signs of IR. The higher concentration of FFA in the body reduces the phosphorylation of tyrosine of IRS-1 and affects the expression of downstream PI3K. ALA effectively reduced the contents of insulin and FFA in diabetic rats, which was consistent with a previous study (Ko et al., 2021). As the time given for HFD increases, the weight of the brain tissue tends to decrease (Lyn-Cook et al., 2009), which is consistent with the results of this study.

AKT plays a critical role in the transmission of insulin signals and regulates cell growth, survival, and metabolism. When IR occurs, insulin signal transmission is inhibited (Huang et al., 2018). In the present study, ALA increased phosphorylation of AKT in the hippocampus and cortex of T2DM rats. Additionally, ALA increased GSK-3β phosphorylation (inactive state) expression in the hippocampus of T2DM rats. The insulin signal pathway of the brain in patients with AD was impaired and the activity of GSK-3β was significantly increased; the activation of GSK-3β in the hippocampus led to phosphorylation of Tau proteins in the T2DM rodent, which further affected memory impairment (Dey et al., 2017). Although the phosphorylation of Tau protein in the ALA treatment group had a tendency to decrease, there was no significant difference in Tau protein between the hippocampus and cortex in this study. Nonetheless, IDE is responsible for the degradation of insulin and a major enzyme that breaks down Aβ in the brain. However, the affinity of insulin for IDE is greater than Aβ, which means that insulin and Aβ will compete with each other for IDE. As a result, the occurrence of hyperinsulinemia may result in the accumulation of Aβ (Kazkayasi et al., 2018). Our results showed that the IDE expression of the hippocampus or cortex of HFD/STZ-induced T2DM rats was greater than the other groups, not only in line with the literature results but also in line with the serum insulin results of this study. Our above findings suggested that ALA alleviates IR of the brain by modulating the IRS-1-insulin signal pathway in HFD/STZ-induced T2DM rats.

Administration of ALA as a dietary supplement in AD patients with DM improved cognitive decline over 16 months on mini-mental state examination scores (Fava et al., 2013). In the present study, ALA improved the memory and learning ability of HFD/STZ-induced T2DM rats. For the passive avoidance test, the consequence of T2DM rats staying in the bright room for a short time was consistent with the results of Firouzjaei et al. (2014). Moreover, in animal models of T1DM, T2DM, AD, and HFD-induced metabolic disorders, on the last day of a space exploration experiment using the WMW protocol, the animals took longer to complete the task than the normal group (Biessels et al., 1998; Cordner and Tamashiro, 2015; Kang et al., 2017). Our results similarly showed that the ALA groups had reduced exploration times. Based on the above results, it is concluded that ALA might modulate in improving the learning memory of T2DM rats.

Long-term potentiation is a memory formation mechanism that relies on the enhancement of synapses. When the NMDAR channel is opened, Ca^2+^ enters the synapse and activates the cascade reaction of calmodulin and CaMKII, and LTP starts to work (Nicoll, 2017). Activated CaMKII can also trigger synaptic plasticity (Wang et al., 2014). NMDAR is the receptor and ion channel protein of postsynaptic glutamate, which is very important for synaptic plasticity and memory function (Crump et al., 2001). It has been verified that the accumulation of Aβ can affect the LTP process and impair memory (Pozueta et al., 2013). Unfortunately, the results of this study showed that the two subunit results of NMDA did not reach significant differences among the groups. Preceding studies using HFD-induced obesity accompanied by IR impaired neuronal survival, affected learning memory, and impaired brain synaptic plasticity (Liu et al., 2017). The results of calmodulin and CaMKII in this study showed that ALA treatment could improve obesity and the damage caused by IR to synaptic plasticity. Additionally, the storage of learning and memory is determined by the postsynaptic density. PSD-95 is the most abundant scaffold protein in the postsynaptic density and plays an important role in synaptic plasticity (Chen et al., 2011). CREB induced LTP formation (Silva et al., 1998). Administration of ALA significantly increased the expression of CREB and PSD-95 in the hippocampus and cortex in this study. It is assumed that ALA might increase synaptic plasticity and enhance the effect of learning memory.

## Conclusion

Administration of ALA for 13 weeks significantly improved the abnormal lipid metabolism and IR of brain in HFD/STZ-induced T2DM rats. Results of passive avoidance and the MWM test showed that ALA ameliorated the learning memory abilities of T2DM rats. Western blot analyses of insulin signaling pathways showed that ALA can improve insulin sensitivity in the brain of T2DM rats, activate insulin signaling pathways, and inhibit the activity of GSK-3β. Expression levels of the LTP pathway downstream proteins showed that ALA can improve synaptic plasticity in T2DM rats. Our results suggested that ALA alleviates HFD/STZ-induced brain IR in T2DM rats to improve learning memory. However, the detailed mechanism of ALA to improve LTP has yet to be clarified in the future.

## Data Availability Statement

The original contributions presented in the study are included in the article/supplementary material, further inquiries can be directed to the corresponding author.

## Ethics Statement

The animal study was reviewed and approved by The Institutional Animal Care and Use Committee of National Taiwan Normal University.

## Author Contributions

C-YK and S-CS participated in the design of the study and writing the protocol. R-ST carried out all experiments. C-YK, J-HX, YL, JW, W-CH, and S-CS conducted kinds of literature searches and data analyses and writing drafts of the manuscript. All authors contributed to the article and approved the submitted version.

## Conflict of Interest

The authors declare that the research was conducted in the absence of any commercial or financial relationships that could be construed as a potential conflict of interest.

## Publisher’s Note

All claims expressed in this article are solely those of the authors and do not necessarily represent those of their affiliated organizations, or those of the publisher, the editors and the reviewers. Any product that may be evaluated in this article, or claim that may be made by its manufacturer, is not guaranteed or endorsed by the publisher.
